# Accuracy of 3D Ground Radio Station Location by a Single Unmanned Aerial Vehicle (UAV) as a Function of an Increasing Number of Received Signal Strength Indicator (RSSI) Measurements †

**DOI:** 10.3390/s25175452

**Published:** 2025-09-03

**Authors:** Jaroslaw Michalak

**Affiliations:** Institute of Communications Systems, Faculty of Electronics, Military University of Technology, Kaliskiego St. No. 2, 00-908 Warsaw, Poland; jaroslaw.michalak@wat.edu.pl

**Keywords:** UAV, Rice, location, 3D space, RSSI, Kalman

## Abstract

The article presents the results of simulation studies assessing the potential value of increasing the accuracy of radio signal source localization as a function of the increasing number of measures performed by a simple UAV (omnidirectional antenna, low flight altitude) in the Rice channel conditions and 3D space. The comparison was made for Range-Based localization methods such as Min–Max, Multilateration, and Nonlinear Regression with an additional assessment of the impact of Kalman filtering. It is estimated that, depending on the adopted localization method, thanks to the use of a large number of measurements performed by the UAV, one can count on a 2 to 6 times increase in localization accuracy in relation to the variant limited by measurements. The above is a good prognosis for the multi-task use of COTS UAV.

## 1. Introduction

Recently, we can observe a significant increase in the usefulness and effectiveness of using small and, at the same time, relatively inexpensive Unmanned Aerial Vehicles (UAVs) and small drone systems (sUAVs) in the implementation of civil and military tasks (e.g., science, ecology, agriculture, transport, communication, reconnaissance, defense and attack [1,2,3,4,5,6,7]. At the same time, note that the development of countermeasure systems (and the increasing range of effective operation of these devices and systems) forces the verification of the method of carrying out missions by small UAVs, including reconnaissance missions. Not all previously used methods to increase the accuracy of localizing the position of the radio signal source are applicable in the special conditions (e.g., locating the position of a radio signal source in an inaccessible, new or even hostile area). In this context, the possibility of collecting a large number of bearings at a considerable distance from its source takes on a new meaning and may be the basis for changing the method of carrying out the task of localizing the source of the radio signal.

Cheap, commercially produced UAVs that are easily available and easy to operate also enjoy growing popularity. Here, it is worth mentioning the following applications as an example:

1. Support of radio network coverage (retransmission points);
2. Temporary communication in crisis areas (voice communication, access to databases);
3. Infrastructure, border inspection tasks, and intelligence gathering;
4. Crop Management;
5. Terrain Monitoring, Mapping, and Cartography.

The rapid development of artificial intelligence [8,9,10] and tools (Hardware Defined Radio (HDR) and Software Defined Radio (SDR)) that allow the autonomous operation of these UAVs [11,12] is also significant in the area discussed.

The control of space at low altitudes above ground is becoming particularly popular and useful due to the rapidly growing number of UAVs performing tasks in these spaces. This also results in the need for 3D localization and is the starting point for the construction of, e.g., automated defense systems, especially in the conditions of the expected significant number of small UAVs and the expected quick reaction of the defense system.

This article attempts to assess the potential degree of increasing the accuracy of localizing the position of the radio signal source in 3D space as a function of the growing number of Reference Points (RP, number of RSSI measurement points performed by the UAV during the flight (up to hundreds)), assuming that the executive device is a Commercial of The Shelf (COTS) UAV using Range-Based methods. The contribution of this article to the field of technical knowledge is as follows:

1. The concept of using cheap COTS UAVs with a simple antenna system and low computing power to implement the function of locating radio signal sources (own and/or foreign);
2. Proposal for implementing the locating function while simultaneously implementing other tasks, such as those related to ensuring, e.g., the connectivity of one’s own radio network;
3. Assessment of the scope of application effectiveness of using Kalman filtering to smooth Received Signal Strength Indicator (RSSI) sample level fading;
4. Comparative assessment of the potential for increasing locating accuracy as a function of the increase in the number of RSSI measurement points performed during the UAV flight;
5. Opening a discussion on the prospect of using traditional, expensive (usually stationary) radio direction finders.

This paper is an extended version of our work presented at [13].

The next chapter reviews the literature on the subject, after which the assumed network structure is presented. In Section 4, the assumptions for the radio channel model are briefly presented, and in Section 5, the tested localization methods are characterized. The results of the simulation evaluation are collected in Section 6.

## 2. Related Works

The issue of locating the position of a radio signal source is generally a major technical task. The most popular application area on the civilian market is, of course, mobile telephony, where organizational and technical conditions have influenced the use of popular subscriber location methods such as Time of Arrival (TOA), Time Difference of Arrival (TDOA), Angle of Arrival (AoA) and Phase of Arrival (POA, ref. [10,14,15]).

In [16], a methodology for locating mobile phones based on crowd-sensing and Received Signal Strength (RSS) measurement was proposed. The authors of [17], as a result of the conducted experiment assessing the accuracy of locating the source using the TDoA, AoA and RSS methods used simultaneously, prove the advisability of increasing the number of sensors. However, it should be noted that in the discussed experiment, due to the nature of the system, the number of these sensors was relatively small (up to 9). An interesting approach to increase the accuracy and speed of source localization may be the cooperative approach, based on a group of UAVs (e.g., ref. [18]). However, this issue is beyond the scope of this article. In [19], the authors investigated the effectiveness of source localization based on RSS in multipath conditions, using a Hidden Markov Model (HMM) within a Bayesian learning framework for signal processing. However, the solution requires relatively high computing power. In the conditions of dynamic operations, we cannot assume the availability (validity) of spectral maps, the creation of which was assumed in [20,21]

From the above, it can be concluded that simple Range-Based localization methods, based on the analysis of the received signal strength indicator (e.g., ref. [22,23,24]) are still in the spectrum of interest.

Despite the obvious lower accuracy of such a method, the advantage is the possibility of using simple antenna systems and the low computing power of the analysis systems. This is the situation we encounter in practice in the dynamically developing applications of relatively cheap, commercial sUAVs performing tasks at low altitudes (approx. 100 m above ground level (AGL)). An increase in source location can be obtained by filtering the recorded RSS and/or increasing the number of measurement points, which in the case of UAVs is a relatively easy task (refs. [25,26]).

In the author’s earlier studies, an attempt was made to assess the degree of increasing the accuracy of the radio signal source position assessment in the channel in 2D space by assessing the potential for increasing the accuracy of selected RSSI-based methods, depending on the selected method, at a level of up to 10 times.

## 3. The Network Structure

**Figure 1:**
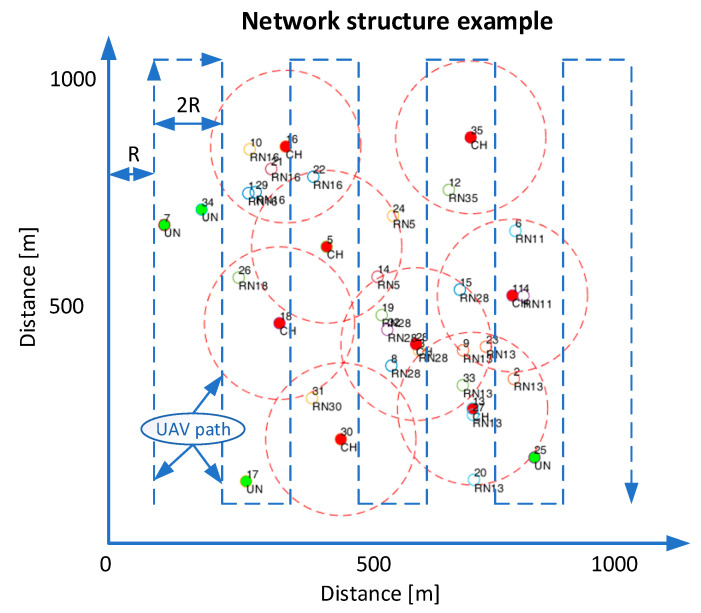
An example of the network structure with a marked UAV route (dashed line). R—UAV turn radius, Points: red—Cluster Head (CH), Green—a Node outside the network (disconnected), No color—Regular Node (RN, operated by a CH).

The effectiveness of increasing the number of RPs in the 3D space Rice channel, which was parameterized according to the literature recommendations, e.g., refs. [27,28]. The network structure is shown in Figure 1.

The structure presented in Figure 1 shows an example of a so-called clustered network, quite popular in some applications. This is a locally centralized, often self-organizing network, where, as a result of node election, some of them assume the role of manager of a group of nodes (cluster, Cluster Head in the figure), while the remaining ones are regular nodes (RNs). RNs are “served” by their own and/or neighboring CHs. One of the challenges of such an organized network is ensuring its full connectivity, meaning that each network node can communicate with every other node in the network. Methods for achieving this connectivity are beyond the scope of this paper, but one approach is to use a UAV as a network monitoring tool (the UAV route in the studied system is marked with a dashed line and depends primarily on the network location ID and the radio range of individual nodes).

The UAV route, which supports the radio network, has been planned to ensure full connectivity of the structure of ground nodes (connectivity = 1, ref. [29]). It is worth noting that the UAV does not directly and exclusively perform the task of tracking the position of the radio signal source. This task is performed as a secondary task to the implementation of another goal, and therefore the UAV route is not optimized to minimize the value of the location error. The drone is moving around the area of a square with a side of L = 1000 m.

## 4. The Radio Channel Model

When analyzing channel models suitable for low-flying UAVs, with typical suburban and rural development, the most appropriate may seem to be the Rice dispersive channel model [28], in which, in addition to the reflected paths, there is a dominant direct path (LOS, e.g., ref. [30]). In such conditions, the signal fading depth is moderate.

The Rice channel is defined by 2 parameters:

1. The factor K reflecting the ratio of the direct path power to the sum of the reflected path powers (the higher the value, the smaller the depth of the fading): $K=\frac{\vartheta^{2}}{2\partial^{2}}$.
2. The total value of the received power as $\Omega =\vartheta^{2}+2\partial^{2}$.

Hence, the power of the received and reflected signal will be written as follows:

(1) $$\vartheta^{2}=\frac{K}{1+K}$$

(2) $$\partial^{2}=\frac{\Omega }{2(1+K)}$$

and the probability density function as:

(3) $$f\left(x\right)=\frac{2\left(K+1\right)x}{\Omega }\exp\left(-K-\frac{\left(K+1\right)x^{2}}{\Omega }\right)I_{0}\left(2\sqrt{\frac{K\left(K+1\right)}{\Omega }x}\right).$$

Based on the analysis conducted by [31], the suburban and rural terrain and the K=14 coefficient were assumed.

According to the author, this type of terrain is most common for typical communication network scenarios supported by UAVs. These include residential and industrial areas. This is, of course, one possible scenario. The lower the flight altitude and the relatively higher the built-up area (or, for example, mountainous terrain), the type of propagation will adopt characteristics closer to the Rayleigh model or will vary depending on the UAV’s flight altitude. Previous articles examined both the effect of changing the Tracking Base (TB) parameter value [31] and changing the K factor, including testing the accuracy of tracking in Rayleigh channel conditions. A decrease in tracking accuracy can be observed with deeper fades. Changing the TB value (if the RSSI sample count is maintained) does not significantly affect tracking accuracy. The Doppler shift was neglected in the channel model, assuming that the UAV movement speed is low. The antenna pattern was also not analyzed, assuming its omnidirectional pattern.

## 5. Location Methods

A brief description of the following Range-Based localization methods will be presented below: Min–Max, Least-Squares and Nonlinear Regression.

### 5.1. Min–Max

The Min–Max method has been described, e.g., in [32,33]. It involves collecting RSSI levels from RPs located at positions $\left(x_{i},y_{i}\right)$ where i refers to the position number. Each RSSI value is then converted into a distance from the signal source, $d_{i}$, using a specific path attenuation equation. Subsequently, for each $d_{i}$, squares (in 2D scenario) within the circles of the estimated ranges are defined. The source’s position is assumed to be at the center of the common area bounded by $x_{min},x_{max},y_{min},y_{max}$.

#### 5.1.1. Min–Max Localization Method for 3D Space and 3 RP

**Figure 2:**
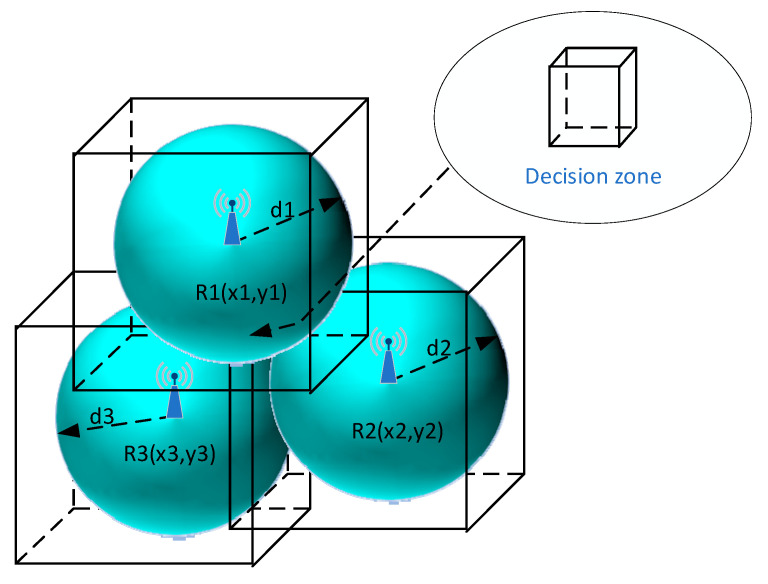
Visualization of the Min–Max method in 3D space and 3 Reference Points.

For 3D and 3 reference points, it can be expressed in Equations (4)–(9). The center of this common area $\left(x_{t},\;y_{t},\;z_{t}\right)$ is determined by Equations (10)–(12) (Figure 2).

(4) $$x_{min}=max(x_{1}-d_{1},\;x_{2}-d_{2},\;x_{3}-d_{3})$$

(5) $$x_{max}=min(x_{1}+d_{1},\;x_{2}+d_{2},\;x_{3}+d_{3})$$

(6) $$y_{min}=max(y_{1}-d_{1},\;y_{2}-d_{2},\;y_{3}-d_{3})$$

(7) $$y_{max}=min(y_{1}+d_{1},\;y_{2}+d_{2},\;y_{3}+d_{3})$$

(8) $$z_{min}=max(z_{1}-d_{1},\;z_{2}-d_{2},\;z_{3}-d_{3})$$

(9) $$z_{max}=min(z_{1}+d_{1},\;z_{2}+d_{2},\;z_{3}+d_{3})$$

(10) $$x_{t}=\frac{\left(x_{min}+x_{max}\right)}{2}$$

(11) $$y_{t}=\frac{\left(y_{min}+y_{max}\right)}{2}$$

(12) $$z_{t}=\frac{\left(z_{min}+z_{max}\right)}{2}$$

#### 5.1.2. Min–Max Localization Method for 3D Space and Multiple RP

In the case of multiple RPs, we can rewrite Equations (4)–(9) in the form (13)–(18) accordingly.

(13) $$x_{min}=max(x_{1}-d_{1},\;x_{2}-d_{2},\;x_{3}-d_{3},\;\ldots ,\;x_{n}-d_{n})$$

(14) $$x_{max}=min(x_{1}+d_{1},\;x_{2}+d_{2},\;x_{3}+d_{3},\;\ldots ,\;x_{n}+d_{n})$$

(15) $$y_{min}=max(y_{1}-d_{1},\;y_{2}-d_{2},\;y_{3}-d_{3},\;\ldots ,\;y_{n}-d_{n})$$

(16) $$y_{max}=min(y_{1}+d_{1},\;y_{2}+d_{2},\;y_{3}+d_{3},\;\ldots ,\;y_{n}+d_{n})$$

(17) $$z_{min}=max(z_{1}-d_{1},\;z_{2}-d_{2},\;z_{3}-d_{3},\;\ldots ,\;z_{n}-d_{n})$$

(18) $$z_{max}=min(z_{1}+d_{1},\;z_{2}+d_{2},\;z_{3}+d_{3},\;\ldots ,\;z_{n}+d_{n})$$

The center of the common region $\left(\hat{x_{t}},\hat{y_{t}},\hat{z_{t}}\right)$ is defined as above by Equations (10)–(12). The location error for a single pass with n RP can be written as:

(19) $$Est.Pos.\;Error=\sqrt{{\left(x-\hat{x_{t}}\right)}^{2}+{\left(y-\hat{y_{t}}\right)}^{2}+{\left(z-\hat{z_{t}}\right)}^{2}},$$

where *x*, *y*, *z*—the actual coordinates of the signal source.

For many measurement repetitions n (many passes and channel realizations), the average position location error will be written as:

(20) $$Est.Pos.\;Error=\frac{\sum_{l=1}^{n}\sqrt{{\left(x-\hat{x_{i}}\right)}^{2}+{\left(y-\hat{y_{i}}\right)}^{2}+{\left(z-\hat{z_{l}}\right)}^{2}}}{n},$$

### 5.2. Multilateration

**Figure 3:**
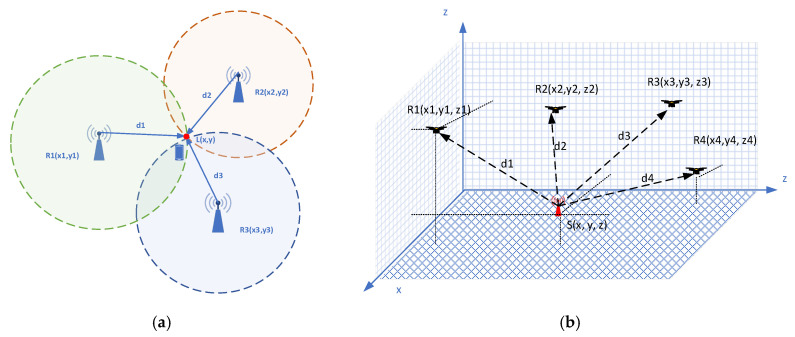
(**a**) 2D Trilateration, (**b**) 3D Trilateration.

The mathematical foundations of several Range-Based localization methods for the 2D space are described in [33]. At this point, it is worth defining the method of multi-point estimation of the emitting source position in a 3D space. To obtain comparative results, the so-called COmplexity-reduced 3D trilateration Localization Approach (COLA), ref. [34] adapted to the multipoint case was chosen. In the simulation model, Anchor Points $\left(x_{i},y_{i},z_{i}\right)$ were assigned to the UAV positions at the RP and the estimated distance from the signal source $d_{i}$. was assigned to each of these positions accordingly. In Figure 3, for clarity, the described situation is shown in the example of 3 RP.

### 5.3. Nonlinear Regression

Nonlinear Regression (NR) is a method used to find a nonlinear model that describes the relationship between a dependent variable and a set of independent variables. Unlike traditional linear regression, which only estimates linear models, Nonlinear Regression can estimate models with complex relationships between the variables. This is performed using iterative estimation algorithms. For these algorithms to work correctly, it is crucial to accurately define the function representing the relationship and to choose appropriate initial values.

In essence, Nonlinear Regression fits a curve or line to a dataset by creating a function that matches an equation to the data. The model’s fitness is assessed by calculating the sum of squares, which measures the differences between the mean and each data point. The goal is to minimize the sum of squares using iterative numerical methods. The principle of least squares is applied to achieve the most accurate parameter estimates by quantifying how much each observation deviates from the mean of the dataset.

In this context, the MATLAB (R2025a (25.1.0.2943329)) Nonlinear Regression function was utilized to estimate the location of the radio signal source. The model function modelfun = @ (P, A) (abs (P (1) − A (:, 1)).^ 2 + abs (P (2) − A (:, 2)).^ 2+abs (P (3) − A (:, 3)).^ 2).^ (1/2), and the starting point for finding the optimal solution (the position of the target station) was established at the center of the monitored 3D area (P0 = [500, 500, 50], A is the UAV position vector). To minimize the model error, the ‘fitnlm’ function was used [35].

This is a function of the Statistics and Machine Learning Toolbox. Its use was performed in the following sequence:

1. Defining a function representing the nonlinear model (the modelfun power function given above)
2. Providing data (the UAV position vector and its associated RSSI vector)
3. Providing the initial values of the model parameters (the center point of the P0 space)
4. Running the “fitnlm” function iterations to achieve the minimum sum of squared residuals of the model (with input parameters: A, P0, modelfun, and d as estimated distances to the signal source)
5. Reading the estimated position of the signal source

## 6. Simulation Results

### 6.1. System Parametrization

**Figure 4:**
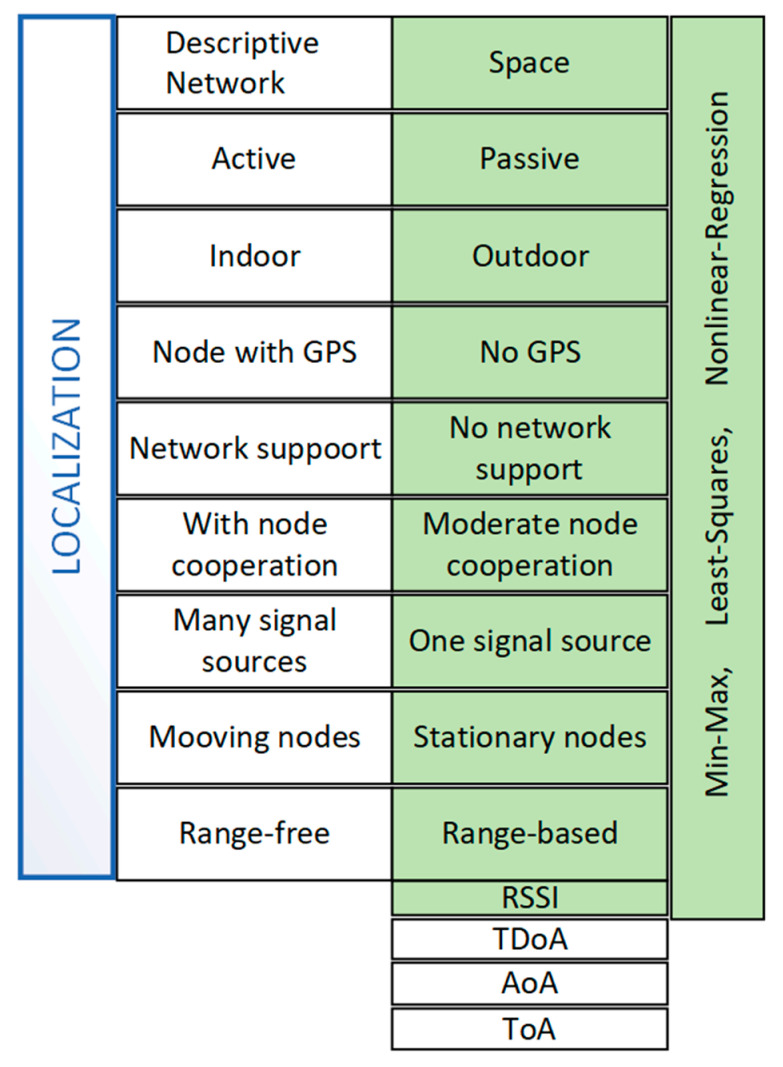
Simulation conditions.

Figure 4 shows the most important conditions and scope of the simulation, while basic information on the parameterization of the simulation model is presented in Table 1.

As can be seen, the radio network was assumed to be spatially distributed (3D space, outdoors). The nodes were assumed to be passive, meaning they did not support the UAV (do not cooperate with it) in the tracking task (the UAV only received the signal from the transmitter and recorded the RSSI level). They did not have a GPS receiver, so they could not independently determine their geographical location. It was also assumed that the radio network nodes did not move. For the simulation, it was assumed that a single signal source was being tracked and that simple RSSI-based methods were used.

The Tracking Base (Table 1) value informs about the distance between subsequent measurements recorded by the UAV. The effect of changing this value was analyzed during previous studies [33]. Here, the studies were conducted by averaging the results for 4 different UAV flight radii R (Figure 1) and for 100 random channel realizations. Two variants of the flight altitude were assumed: the case in which the UAV flies at a constant height of H = 100 m AGL along the route marked in Figure 1 with a dashed line, and the case when the UAV evenly changes its height in the range from 0 to 100 m AGL. RSSI measurements are collected with a resolution of 10 m (Tracking Base). In the case of the Rice channel, the option of filtering the signal measured with the Kalman filter was also used.

### 6.2. Results

#### 6.2.1. Free Space Path Loss Channel

This part of the experiment was conducted for comparative purposes, as a reference point for the results obtained under Rice channel fading conditions. This is also important because the adopted simulation simplifications (discussed, for example, in Section 7) mean that the source location accuracy is not as precise as in applications employing additional technical and organizational measures.

**Figure 5:**
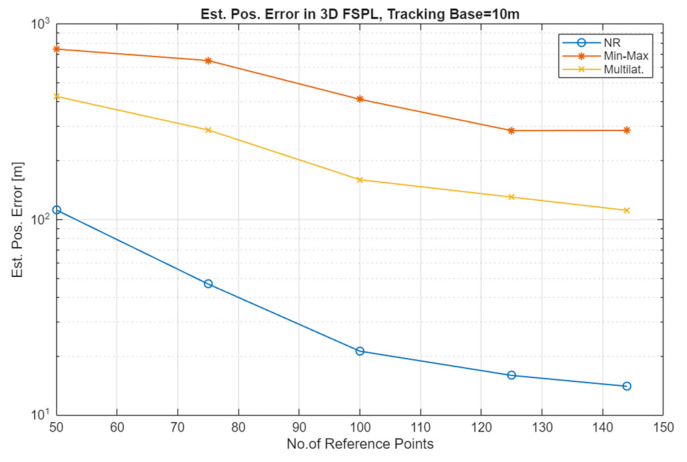
Estimated position error in the FSPL channel for a constant value of the TB and an increasing number of RP (variable UAV altitude (0–100 m AGL), one repetition).

**Figure 6:**
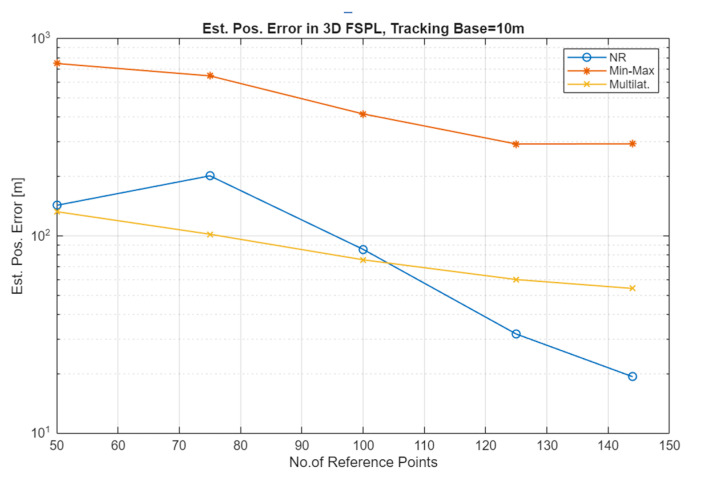
Estimated position error in the FSPL channel for a constant value of the TB and an increasing number of RP (constant UAV altitude (100 m AGL), one repetition).

**Figure 7:**
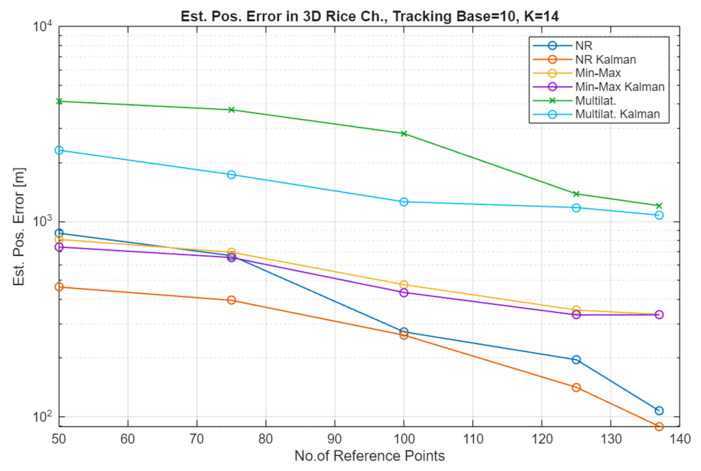
Estimated position error in the Rice channel for a constant value of the TB and an increasing number of the RP (variable UAV altitude (0–100 m AGL), average of 100 repetitions).

**Figure 8:**
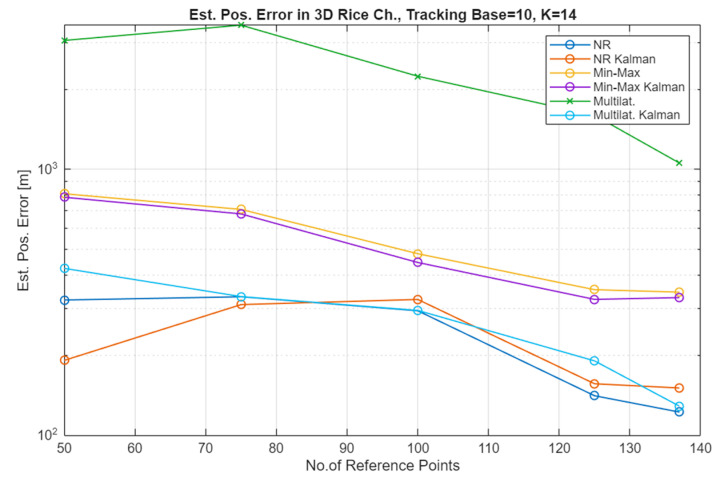
Estimated position error in the Rice channel for a constant value of the TB and an increasing number of the RP (constant UAV altitude (100 m AGL)), average of 100 repetitions.

The simulation results for the Free Space Path Loss (FSPL) channel (comparative plots) and for the Rice channel are presented in Figure 5, Figure 6, Figure 7 and Figure 8.

Analyzing the results for the FSPL channel, it can be seen that depending on the localization method, one can count on improving its accuracy by about 2 to 6 times. For the FSPL channel, no significant dependencies of the results on whether the UAV moved at a constant height or whether this height was variable were observed.

#### 6.2.2. Rice Channel

In the case of the Rice channel, an improvement in localization accuracy can be observed as a function of the increasing number of bearing points, depending on the localization method, in a ratio of 2 to 5, so slightly worse than for the FSPL channel. It can also be stated that the filtering of the received signal is applicable for a small number of RP, i.e., with their increasing number, the result analysis systems can be simplified.

## 7. Discussion

This study is closely related to the author’s earlier work on determining the route of a simple, multirotor UAV performing the task of ensuring the connectivity of the radio communication network. This type of UAV, while performing measurements of the level of radio signals, can simultaneously perform other functions that are related to, for example, spectrum monitoring and, in this case, locating unidentified signal sources. The results of the locating accuracy presented above should not be assessed directly (as an absolute value) because the study does not consider the features and method of operation of UAVs typical for the implementation of locating tasks. Hence, it is only necessary to pay attention to the percentage (multiple) gain in increasing the locating accuracy under the assumed conditions. The task of maximizing the locating accuracy may use additional technical procedures (e.g., AI-based filtering, model calibration, UAV network and data fusion) and organizational procedures (e.g., UAV network, position patterns, established UAV routes [36,37,38,39]). Under the assumed conditions, depending on the method, the effectiveness of Kalman filtering is especially visible with a small number of measurement points (70 to 100, which in the simulation scenario means a flight distance of 700 to 1000 m). I would explain this by the fact that with a larger number of RP, the effect of averaging the final result becomes increasingly effective.

Regarding the computational complexity of the analyzed methods, based on preliminary estimates, it is worth noting that RSSI-based methods are inherently relatively simple. The article does not precisely determine the computational complexity of the algorithms, but it is estimated that:

1. The Min–Max method, being a geometric method, is the fastest solution (least computationally complex) and can be implemented in real time, but is the least accurate. This computational complexity can be expressed as O(N), where N is the number of measurement points.
2. The approach using Nonlinear Regression may be the most accurate, but it has the highest computational requirements in the group discussed, which can be expressed as O(N × I × p), where i is the number of iteration steps for each measurement point and p is the number of parameters. In offline applications, it is more suitable.
3. The compromise approach in the considered group seems to be the multilateration method (here based on the COLA algorithm), offering average accuracy and computational complexity estimated on the Min–Max complexity level.

## 8. Conclusions

As can be seen in Figure 5 and Figure 6, which present the results of the localization accuracy evaluation in the FSPL channel compared to the results in the Rice channel (Figure 7 and Figure 8), the source localization accuracy in the channel with signal fading is significantly worse. The results vary for different localization methods and paths. In general, it can be estimated that the localization error increases at least four-fold.

The conducted assessment of the potential degree of improvement of the accuracy of localizing the radio signal source in 3D space as a function of the increasing number of Reference Points was carried out using the simulation method. It was assumed that the COTS UAV performs a mission at an altitude of up to 100 m AGV, realizing the bearing using the Min–Max, Multilateration, and Nonlinear Regression methods. The assessment was made by assuming the Rice propagation channel with optional Kalman filtering. It is estimated that with the increase in the number of measurement points, one can count on a several-fold increase in localization accuracy, and the use of Kalman filtering is helpful, especially with a small number of the Reference Points (to ab. 70 or 100 in dependance of the location method). Among the studied methods, the nonlinear regression method is the most promising for use in various scenario conditions. Another promising research area in this field is the evaluation of the effectiveness of artificial intelligence algorithms in the filtration process of channel distortions.

## Figures and Tables

**Table 1:** Simulation parameters.

Parameter	Value
Channel	FSPL, Rice
K factor (Rice)	14
Noise variance	1
Number of UAVs	1
Number of RP	362, 295, 204, 144
Tracking Base [m]	10
UAV flight radius [m]	100, 150, 200, 250
UAV flight altitude [m]	100 or 0 to 100

## Data Availability

The data presented in this study are available on request.
